# TLR4 Agonist Combined with Trivalent Protein JointS of *Streptococcus suis* Provides Immunological Protection in Animals

**DOI:** 10.3390/vaccines9020184

**Published:** 2021-02-22

**Authors:** Zhaofei Wang, Mengting Guo, Licheng Kong, Ya Gao, Jingjiao Ma, Yuqiang Cheng, Henan Wang, Yaxian Yan, Jianhe Sun

**Affiliations:** 1 Shanghai Key Laboratory of Veterinary Biotechnology, Shanghai Jiao Tong University, Shanghai 200240, China; wzfxlzjx@sjtu.edu.cn (Z.W.); guomengting1994@sjtu.edu.cn (M.G.); klc0713@163.com (L.K.); ele.gaoya@sjtu.edu.cn (Y.G.); majingjiao@sjtu.edu.cn (J.M.); 1987lccyq@163.com (Y.C.); hawang@sjtu.edu.cn (H.W.); yanyaxian@sjtu.edu.cn (Y.Y.); 2 Key Laboratory of Urban Agriculture (South), Ministry of Agriculture, Shanghai Jiao Tong University, Shanghai 200240, China

**Keywords:** *Streptococcus suis*, JointS, zebrafish, mice, piglet, subunit vaccine

## Abstract

*Streptococcus suis* (*S. suis*) serotype 2 (SS2) is the causative agent of swine streptococcosis and can cause severe diseases in both pigs and humans. Although the traditional inactive vaccine can protect pigs from SS2 infection, novel vaccine candidates are needed to overcome its shortcomings. Three infection-associated proteins in *S. suis*—muramidase-released protein (MRP), glyceraldehyde-3-phosphate dehydrogenase (GAPDH), and DLD, a novel putative dihydrolipoamide dehydrogenase—have been previously identified by immunoproteomic assays. In this study, the effective immune protection of the recombinant trivalent protein GAPDH-MRP-DLD (JointS) against SS2, SS7, and SS9 was determined in zebrafish. To improve the immune efficacy of JointS, monophosphoryl lipid A (MPLA) as a TLR4 agonist adjuvant, which induces a strong innate immune response in the immune cells of mice and pigs, was combined with JointS to immunize the mice. The results showed that immunized mice could induce the production of a high titer of anti-*S. suis* antibodies; as a result, 100% of mice survived after SS2 infection. Furthermore, JointS provides good protection against virulent SS2 strain infections in piglets. Given the above, there is potential to develop JointS as a novel subunit vaccine for piglets to prevent infection by SS2 and other *S. suis* serotypes.

## 1. Introduction

*Streptococcus suis* (*S. suis*) is an important invasive swine pathogen associated with a wide range of diseases in pigs, including septicemia, pneumonia, meningitis, endocarditis, and arthritis [1,2,3]. At present, 35 serotypes (types 1 to 34 and 1/2) have been identified on the basis of their capsular polysaccharides. Serotype 2 (SS2) is the most pathogenic and prevalent type in diseased pigs in most countries [4,5]. SS2 is also recognized as an important zoonotic pathogen since it has caused severe diseases and even death in humans, especially in those who have frequent contact with diseased pigs or their products. In 1998 and 2005, the cases of human SS2 infection with clinical signs of disease of streptococcal toxic shock syndrome (STSS) were reported in China, resulting in 14 and 38 human deaths, respectively [6,7]. It is therefore necessary and extremely important to advance work in the prevention and control of SS2 infections.

Vaccination is generally recognized as one of the most effective methods of controlling *S. suis* infection, but researchers have not yet found a satisfactory vaccine [1,8]. Most of the traditional whole-cell bacterin lacks antigenicity, which means that the production of antibody to antigen is insufficient to provide protection and/or lacking cross-reactivity. Normally, this deficiency is caused by inadequate heat or formalin processing [8]. Furthermore, bacterins sometimes cause severe side effects, such as hyperthermia, extra connective tissue reactions, or exceptionally specific *S. suis* clinical signs [8]. Therefore, it is necessary to develop an efficient vaccine to protect both pigs and humans from *S. suis* infection. Subunit vaccines based on proteins conserved among serotypes provide a strong cross-immunity, which may be more useful in the field if they protect against challenge with strains of heterologous serotypes [8,9,10]. In this study, three *S. suis* virulence-associated proteins—muramidase-released protein (MRP), glyceraldehyde-3-phosphate dehydrogenase (GAPDH), and dihydrolipoamide dehydrogenase (DLD), which are highly conserved in SS2, SS7, and SS9—were considered subunit vaccine candidates [11,12]. MRP is a virulence-associated protein, but immunization with MRP alone conferred poor protection against challenges with SS2 strains [13]. It is exciting that combining MRP with an extracellular factor (EF) significantly improved protective efficacy, increasing the survival rate from 25% to 88.9% [14]. GAPDH is also an adhesion-associated factor and exists in most invasive isolates, including serotypes 1, 2, 7, and 9 [15]. Moreover, the surface-exposed immune evasion protein DLD is a potential vaccine antigen against *Vibrio* and *M. tuberculosis* [16,17]. To date, no research has focused on the protective immunity of DLD in *S. suis*.

To overcome the relatively low immunogenicity of subunit vaccines as a limitation and the induction of desirable immune responses, an adjuvant is required [18]. Vaccine adjuvant refers to a class of substances that play an auxiliary role to non-specifically enhance the body’s specific immune response to antigens [18,19]. For subunit vaccines, the addition of adjuvants can make them produce a more efficient immune response in the body. Currently, toll-like receptor (TLR) agonists have been widely used in vaccine adjuvants [18,20]. Antigen-presenting cells (APCs) are connected with natural and acquired immune responses and are the target of TLR agonists [21]. TLRs can increase antigen uptake by dendritic cells (DCs), thereby reducing the dose of antigen in the vaccine. In addition, they can facilitate antigen processing and the presentation of MHC-I and MHC-II peptides [22].

In this study, based on the advantages of subunit vaccine safety, stability, easier to produce, and good immune effect, we recombine three important protective antigens (GAPDH, MRP, and DLD) of *Streptococcus suis*, termed JointS. A zebrafish immune protection experiment was used to verify the immune protection of the JointS protein against SS2, SS7, and SS9 infections. Further, in order to enhance the immunoprotective efficacy of JointS, a TLR4 agonist combined with JointS was used to evaluate the immune protective ability in mice against *Streptococcus suis* infection. This provides basic experimental data and new ideas for the development of a safe and efficient, general-purpose *Streptococcus suis* subunit vaccine.

## 2. Materials and Methods

### 2.1. Ethics Statement

All the animal experiments were approved on 03 January 2017 by the Institutional Animal Care and Use Committee of Shanghai Jiao Tong University (Approval no. 20170103). They were performed in accordance with the Animal Care and Use Guidelines of the Ministry of Science and Technology of China.

### 2.2. Bacterial Strains and Culture Conditions

The SS2 strain HA9801 was kindly provided by Professor Chengping Lu of Nanjing Agricultural University in China and was isolated in 1998 from a diseased pig with septicemia in the Haian region of China. *Streptococcus suis* serotype 7 (SS7) strains SS−7 and serotype 9 (SS9) strains SS−9 were stored in our laboratory and isolated from diseased pigs between 1998 and 2005 in China [23]. All *S. suis* strains were cultured in Todd Hewitt broth (THB) in a shaker (180 rpm) at 37 °C. The bacterial burden, which was given as the number of colony forming units (CFU) of *S. suis*, was measured in THB agar supplemented by 5% (vol/vol) fresh sheep blood under static conditions at 37 °C. The *Escherichia coli* (*E. coli*) strain BL21, which was used in gene cloning and the production of recombinant proteins, was grown in Luria-Bertani (LB) broth with shaking (180 rpm) at 37 °C.

### 2.3. Gene Amplification and Construction of Expression Vectors

The primers (P1/P2, P3/P4, and P5/P6) used to amplify the genes of *mrp*, *gapdh*, and *dld* from the chromosomal DNA of HA9801 are listed in Table 1. The fused *gapdh*-*mrp*-*dld* gene was constructed as follows. First, the genes of *mrp*, *gapdh*, and *dld* were amplified by using the primer pairs P7/P8, P9/P10, and P11/P12, respectively (Table 1). These PCR productions, with linker sequences (GGAGGTGGAGGTGGA) and overlaps of each gene, were used as a template to generate a fused fragment of *gapdh*-*mrp*-*dld* (*JointS*) using the primer pair P7/P12 (Table 1).

The amplified PCR products were ligated into the pET − 28 an expression vector. The recombinant plasmids were introduced into *E. coli* DH5α, and the clones were selected by growing the cultures on LB agar in the presence of 50 μg/mL kanamycin. After that, the plasmids from positive clones, which were checked by PCR and DNA sequence analyses, were introduced into *E. coli* BL21 (DE3) for expression.

### 2.4. The Expression, Purification, and Antigenicity Identification of GAPDH, MRP, DLD, and GAPDH-MRP-DLD (JointS)

The bacterial cells were cultured in LB broth at 37 °C until the optical density was 0.6 at 600 nm (OD600). After cooling, 1 mM isopropyl-β-D-thiogalactoside was added to induce the production and then shaken at 30 °C for 5 h. The induced cells were washed, resuspended, and homogenized using a sonicator. After centrifugation, the protein was purified using affinity chromatography with Ni-NTA columns (GE Healthcare BioSciences, Pittsburgh, PA, USA). To obtain a recombinant protein for use in the animal experiments, endotoxin was removed using phosphate-buffered saline (PBS) containing 0.1% Triton X-114 (Sigma-Aldrich, St. Louis, MO, USA) to elute protein from the Ni-NTA columns [24]. Sodium dodecyl sulfate-polyacrylamide gel electrophoresis (SDS-PAGE) analyses were performed using the purified proteins.

The antigenicity of GAPDH, MRP, DLD, and JointS was tested by Western blot using the serum obtained from SS2-infected pigs, as described previously [13]. Briefly, the purified proteins were separated on SDS-polyacrylamide gel and transferred to polyvinylidene difluoride membranes. The pig serum samples (1:2000) were used as the primary antibody, and horseradish peroxidase (HRP)-conjugated goat anti-pig immunoglobulin (1:10,000; Sigma-Aldrich) was used as the secondary antibody. The images were collected using a Tanon 5200 imaging system (Tanon, Shanghai, China).

### 2.5. Zebrafish Immunization with JointS and Challenge with S. suis

#### 2.5.1. A 50% Lethal Dose of *S. suis* Determination in Zebrafish

The zebrafish were tested using *S. suis* as described previously [25]. Prior to the inoculation of the fish, the *S. suis* culture (SS2, SS7, and SS9) was collected in the late log phase and washed twice in phosphate-buffered saline (PBS) (pH 7.4). The zebrafish were anesthetized by tricaine methanesulfonate (MS−222) (Hangzhou Animal Medicine Factory, China). Several groups of 15 zebrafish were injected intraperitoneally (i.*p*.) with 50-fold serially diluted suspensions of bacteria in sterile PBS (Table 2, Table 3 and Table 4). The control fish were injected with sterile PBS. The survival rates were recorded daily and then over a period of 7 days post-infection. The 50% lethal dose (LD_50_) values were calculated by the Reed and Muench method [26].

#### 2.5.2. Immunization and Challenge of Zebrafish

The zebrafish were assigned to 15 groups of 20 each. After anesthesia by MS-222, each zebrafish was immunized by an intraperitoneal injection of 25 μL recombinant protein (GAPDH, MRP, DLD, and JointS; 25 μg) or PBS (25 μL, control group). After primary immunization, the same dosage of protein was repeated on day 14 (Supplementary materials Figure S2). During the immunization, the water temperature was held at 25 ± 1 °C. Seven days after the booster immunization, all groups of zebrafish were challenged by intraperitoneal injection with 10-fold LD_50_ of a log-phase culture (25 μL) of SS2, SS7, or SS9. The survival rates were recorded daily and then over a period of 7 days post-infection.

### 2.6. The Cytokine Expression of Immune Cells Induced by TLR Agonists Using qRT-PCR

RAW264.7 cells (ATCC) were maintained in Dulbecco’s modified Eagle medium (DMEM) with high glucose (Gibco, Invitrogen Corp., Carlsbad, CA, USA) supplemented by 10% (vol/vol) heat-inactivated fetal bovine serum (FBS) (Invitrogen). Porcine alveolar macrophages (PAMs) were isolated from piglets as previously described and cultured in RPMI 1640 containing 10% FBS, penicillin, streptomycin, and GlutaMAX (all purchased from Thermo Fisher Scientific, Shanghai, China) at 37 °C with 5% CO_2_ [27]. For cell stimulation, RAW264.7 cells and PAMs were grown in 12-well tissue culture plates at a concentration of 4 × 10^5^ cells/well. Monophosphoryl lipid A (MPLA) or Imiquimod (R837) was reconstituted in sterile, endotoxin-free water at a concentration of 0.5 mg/mL and stored frozen. This stock was serially diluted in the appropriate medium (0.5, 1, and 2 μg for MPLA; 3 and 5 μg for R837) and added to the cell monolayers at 37 °C with 5% CO_2_, the untreated cells served as the control.

After 24 h, total RNA was extracted from the cells by the Trizol method (Takara Biotechnology, Dalian, China), as previously described [28]. The cDNA synthesis was performed using the PrimeScript RT reagent kit (TaKaRa) according to the manufacturer’s instructions. The mRNA levels were measured using a two-step relative qRT-PCR. Murine or porcine *gapdh* gene was amplified as reference genes. The specific primers are shown in Table 5. A qRT-PCR was performed using a SYBR Premix Ex Taq kit (TaKaRa) and an ABI 7500 RT-PCR system. The relative gene expression was calculated using the 2^-(∆Ct)^ method [28]. Levels of IL-6, IL-8, and IFN-γ mRNA were normalized to the mRNA levels of the reference gene; they were then expressed as n-fold increases with respect to the untreated cells (0 μg of MPLA or R837).

### 2.7. Mice Immunization with JointS and Challenge with SS2

Female BALB/c six-week-old mice were purchased from the Experimental Animal Center at Shanghai Jiao Tong University. As shown in Supplementary materials Figure S2, fifteen mice in each group were immunized at 0 days by 50 μg per dose of JointS protein formulated with complete Freund’s adjuvant (FA, Sigma, 200 μg), R837(1 μg), or MPLA (1 μg) for primary and incomplete FA (Sigma, 100 μg), and R837(1 μg) or MPLA (1 μg) for boosts (at 14 and 21 days after the primary immunization). Inactivated SS2 strains (HA9801, 1 × 10^7^ CFU/mouse) formulated with MPLA (1 μg) were performed using the same immune procedure described above. The non-immunized mice served as the control group. All immunizations were performed via the subcutaneous route. Serum samples were collected at 7, 14, and 28 days after the primary immunization, respectively.

After 28 days, lethal doses (5 × 10^8^ CFU/mouse) of SS2 were injected into the abdominal cavities of the mice. Five mice in each group were sacrificed to detect CFU burdens on blood and tissues. The CFU burdens were determined 24 h post-infection in all groups. Each lung or spleen was homogenized in 1 mL of PBS and then serially diluted 10 times in sterile PBS for quantitative culturing on a THB agar plate. All cultures were incubated (37 °C) for 24 h, and the resulting colonies were enumerated as CFU/g (tissues) or CFU/mL (blood). During the study, clinical signs of disease and daily clinical scores were recorded daily until 7 d post-infection. The clinical scores were assigned as follows: 0 = Normal; 1 = Minor illness: inactive and slow to respond, with oculonasal secretions; 2 = Moderate disease: rough hair/coat, narcolepsy, only responsive to repeated stimuli; 3 = Severe disease: ataxia, unaware of surroundings; and 4 = Dead. Recovery was considered a mental state score ≤1. Survival rates were recorded daily and over a period of 7 days post-infection.

### 2.8. Piglets Immunization with JointS and Challenge with SS2

Six four-week-old piglets with no history of *S. suis* infection were purchased from a pig breeding (nursery) farm in Hangtou (Shanghai, China). After one week of adaptation, the piglets were randomly assigned to two groups of three each. One group was immunized intramuscularly by 750 μg of purified JointS emulsified with an adjuvant on day 0 for priming and on day 14 for boosting (Supplementary materials Figure S2). The piglets were mock immunized with PBS emulsified in the same adjuvant, which served as a negative control. On days 0, 7, 14, and 21, blood samples were collected from each piglet by precaval vein bleeding. The sera were separated by centrifugation and stored at −20 °C before further Enzyme-linked immunosorbent assay (ELISA) analysis to determine the antigenicity of JointS. Two weeks after the booster immunization, the immunized and control piglets were intramuscularly challenged by 10 mL (5 × 10^9^ CFU per piglet) of a log-phase culture of SS2 HA9801. The piglets were clinically monitored for seven days after the challenge, including rectal temperatures, body weight, and clinical signs of disease. The daily gain in body weight was calculated as follows: daily gain = (body weight at day x-body weight at day 0)/body weight at day 0. The clinical signs of disease including impaired mental states and claudication were scored. Scores of 0 to 2 were used: 0 = normal; 1 = the piglets avoided movement on the leg and showed signs of lack of coordination visible only after manipulation; 2 = the piglets were reluctant to stand and showed signs of asynergia or lethargy.

### 2.9. Enzyme-Linked Immunosorbent Assay (ELISA)

The blood serum antibody titers in the mice and piglets were determined by an indirect ELISA, as previously described [29]. Briefly, polysorb immunoplates (Nunc, Rochester, NY, USA) were coated overnight at 4 °C with 100 μL inactivated HA9801 (10^8^ CFU/mL) per well or with the proteins GAPDH, MRP, DLD, and JointS (100 μg/mL) in carbonate buffers. The sample serum was used as the primary antibody, and peroxidase-conjugated goat anti-mouse or anti-swine IgG (Sangon Biotech, Shanghai, China) was used as the secondary antibody. The plates were developed with Tetramethylbenzidine (TMB) substrate (Sangon Biotech, Shanghai, China). Absorbance was measured at 450 nm by an ELISA reader (Bio-Tek Instruments, Inc., Winooski, VT, USA).

Commercial ELISA kits (Abcam, Cambridge, UK) were used to evaluate the serum levels of the cytokines of interleukin 6 (IL-6) and IFN-γ of in the mice 28 days after immunization. The cytokine levels were measured in accordance with the manufacturer’s instructions.

### 2.10. Statistical Analysis

In all experiments, data points were plotted using GraphPad Prism 6.01 (GraphPad Software, Inc., San Diego, CA, USA). According to the Shapiro–Wilk normality test, the whole data showed the normal distribution. The statistical significance of changes between groups was assessed with Kruskal Wallis tests followed by Dunn’s post-hoc tests (3 and more groups) or unpaired Student’s *t*-test (two groups). The survival rate was analyzed by a log-rank (Mantel-Cox) test. *p* values are indicated in the figure legends.

## 3. Results

### 3.1. Preparation and Immunoreactivity of GAPDH, MRP, DLD, and JointS

The cloning, prokaryotic expression, and purification of GAPDH, MRP, DLD, and JointS were conducted as described in the Materials and Methods section. The genes of *gapdh*, *mrp*, *dld*, and *gapdh-mrp-dld* were encoded at 41.7, 26.0, 40.5, and 95.5 kDa proteins, respectively. The recombinant proteins were purified by Ni-NTA affinity chromatography and showed good immunoreactivity to convalescent serum against SS2 HA9801 by Western blot analysis (Supplementary materials Figure S1A–D).

### 3.2. The Immune-Protective Effect of Recombinant Protein against Different Serotypes of S. suis in Zebrafish

**Figure 1:**
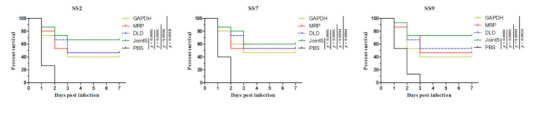
Kaplan–Meier survival curves of vaccinated zebrafish infected with different serotypes of *S. suis*. Seven days after booster immunization, SS2, SS7, or SS9 were injected into the abdominal cavities of the zebrafish. PBS-vaccinated zebrafish infected with *S. suis* served as PBS control groups. Survival rate was analyzed by a log-rank (Mantel-Cox) test.

To determine the immune-protective effects of JointS, the immunization and challenge of zebrafish were performed. Previously, the LD50 of the zebrafish challenged by SS2, SS7, and SS9 was determined (Table 2, Table 3 and Table 4). Two weeks after the booster immunization, all groups of zebrafish were challenged by lethal doses of 50-fold LD_50_ of SS2, SS7, and SS9. All zebrafish in the negative control group died, while 66.7%, 60%, and 73.3% of those vaccinated with JointS survived after being challenged by SS2, SS7, and SS9, respectively (Figure 1). The other single recombinant proteins showed a partial protection effect against *S. suis* infection in zebrafish.

### 3.3. TLR-4 Agonist (MPLA) Stimulates Immune Cells to Produce Cytokines

**Figure 2:**
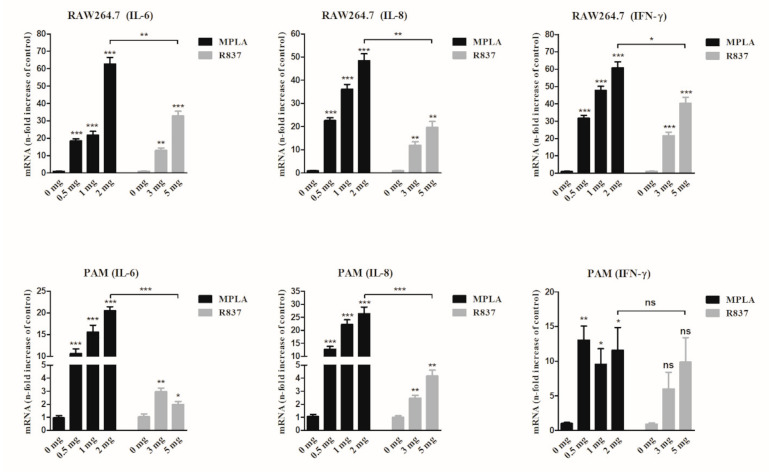
MPLA- and R837-induced cytokine expression in RAW264.7 and PAM cells. IL-6, IL-8, and IFN-γ levels were measured by qRT-PCR. Untreated cells served as the control. Levels of IL-6, IL-8, and IFN-γ mRNA were normalized to the mRNA levels of the reference gene; they were then expressed as n-fold increases with respect to the untreated cells (0 μg of MPLA or R837). Kruskal Wallis test followed by Dunn’s post-hoc test was used to compare the results between the different treated groups and controls. *** *p* < 0.001, ** *p* < 0.01, * *p* < 0.05, and ns (*p* > 0.05). Error bars represent SEM.

In the zebrafish experiment, JointS did not show a dominant protective effect against *Streptococcus suis* infection. To improve the immune effect of the recombinant proteins, two TLR agonists (TLR4-MPLA and TLR7-R837) were compared as candidates for immune adjuvants combined with JointS. The results showed that both MPLA and R837 induced RAW264.7 and PAM cells to produce high levels of innate immune-related cytokines (IL-6, IL-8, and IFN-γ) in a dose-dependent manner (Figure 2). Furthermore, in both murine and porcine cells, compared with R837 (5 μg), relatively low concentrations of MPLA (2 μg) induced significantly higher levels of cytokines, which indicated that MPLA may deliver a better immune response than R837 in murine and porcine cells.

### 3.4. MPLA Combined with JointS to Prevent S. suis Infection in Mice

**Figure 3:**
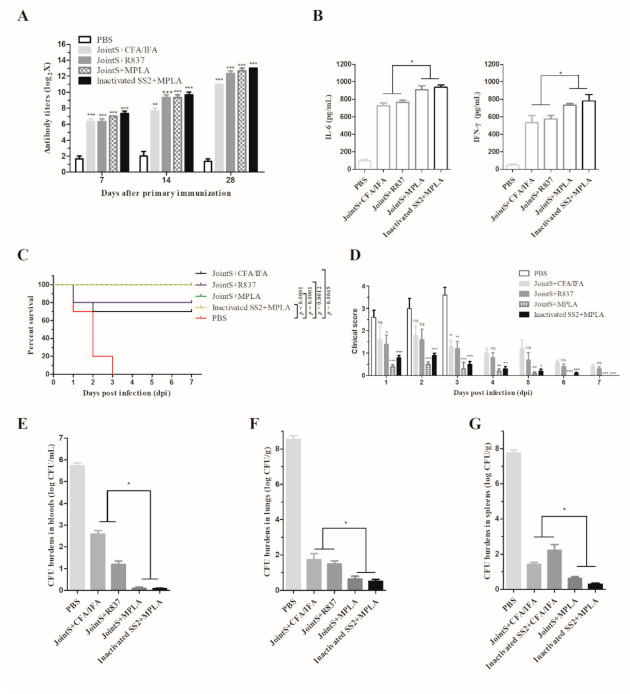
MPLA combined with JointS to prevent *S. suis* infection in mice antibody titer (**A**), cytokine levels (**B**), immune protection rates (**C**), clinical score (**D**), and colony forming unit (CFU) burdens in the blood or tissues (**E**–**G**) in the differently vaccinated groups after challenge by HA9801. The mice were immunized by JointS protein formulated with Freund’s adjuvant (JointS + CFA/IFA), R837 (JointS + R837), or MPLA (JointS + MPLA). The mice immunized by inactivated SS2 (HA9801) strains formulated with MPLA were termed inactivated SS2 + MPLA. The PBS-immunized mice served as the control group. After 28 days, a lethal dose (5 × 10^8^ CFU/mouse) of SS2 was injected into the abdominal cavities of the mice. Survival rates and clinical signs were monitored for seven days post-infection. In (**A**), Kruskal Wallis test followed by Dunn’s post-hoc test showed that the antibody titers in the immunized mice were significantly higher than in the non-immunized mice. In (**B**), an unpaired Student’s *t*-test showed that the cytokine levels in the groups administered antigens formulated with MPLA were significantly higher than in the groups administered JointS formulated with Freund’s adjuvant or R837. In (**C**), survival rates were analyzed by a log-rank (Mantel–Cox) test. In (**D**), Kruskal Wallis test followed by Dunn’s post-hoc test showed that the clinical score in the immunized mice were significantly lower than in the non-immunized mice. In (**E**–**G**), an unpaired Student’s *t*-test showed that the CFU burdens in the groups administered antigens formulated with MPLA were significantly lower than in the groups administered JointS formulated with Freund’s adjuvant or R837. Samples collected from each mouse were tested in triplicate. *** *p* < 0.001, ** *p* < 0.01, * *p* < 0.05, and ns (*p* > 0.05). The error bars represent SEM.

After three times immunization, the immunoreactivity of anti-serum from the vaccinated mice progressively increased. At seven days after the third immunization, the groups of mice vaccinated by JointS showed similar levels of antibody titers compared with the mice vaccinated by inactivated SS2 (Figure 3A). However, the groups of mice vaccinated by JointS and inactivated by SS2 formulated with MPLA showed higher cytokines (IL-6 and IFN-γ) compared with the mice vaccinated with JointS formulated with R837 and Freund’s adjuvant (Figure 3B).

One day after the administration of the challenge infection HA9801, all mice in the non-immunized control group exhibited significant clinical signs, such as ruffled hair coats, narcolepsy, and even unawareness of the surroundings (Figure 3C). After three days, all non-immunized mice died. In contrast, all the mice in the groups vaccinated by JointS or inactivated SS2 formulated with MPLA survived the *S. suis* infection, indicating complete protection (Figure 3C). However, the JointS + MPLA vaccinated mice showed relatively lower clinical scores (i.e., a small amount of oculonasal secretions and transient rough hair) than the mice that had been administered the inactivated vaccine (i.e., narcolepsy and rough hair coat) on the first and second days after infection (Figure 3D). In addition, JointS formulated with R837 and Freund’s adjuvant showed relatively less immune protection (80% and 70%, respectively) compared with the antigen formulated with MPLA (Figure 3C,D).

In both the blood and tissues, the CFU burdens in the vaccinated group were significantly lower than in the nonimmunized group (Figure 3E–G). In the blood, almost no bacteria were detected in the JointS or inactivated SS2-formulated MPLA vaccinated group. In contrast, the CFU burdens remained detectable in the mice vaccinated by JointS formulated with Freund’s adjuvant or R837 (Figure 3E). In addition, in the lung and spleen, the CFU burdens in the groups administered antigens formulated with MPLA were significantly lower than in the groups administered JointS formulated with Freund’s adjuvant or R837 (Figure 3F,G).

### 3.5. Application of JointS in Prevention of S. suis Infection In Piglets

**Figure 4:**
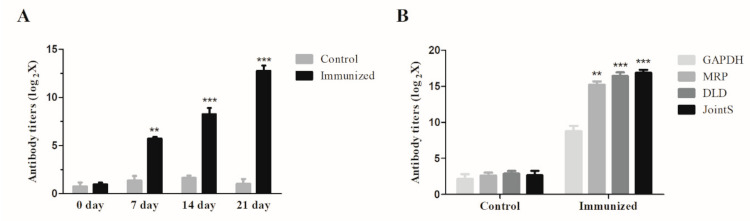
Antibody titers in the vaccinated piglets. Antibody titers were tested by ELISA using inactivated SS2 (**A**) or purified GAPDH, MRP, and DLD proteins (**B**) with immunized sera. Samples collected from each piglet were tested in triplicate. In (**A**), an unpaired Student’s *t*-test showed the antibody titers in the JointS immunized mice were significantly higher than PBS-immunized mice in different time points. In (**B**), Kruskal Wallis test followed by Dunn’s post-hoc test showed the specific antibody titers against GAPDH were significantly lower than MRP, DLD, or JointS. *** *p* < 0.001 and ** *p* < 0.01. The error bars represent SEM.

**Figure 5:**
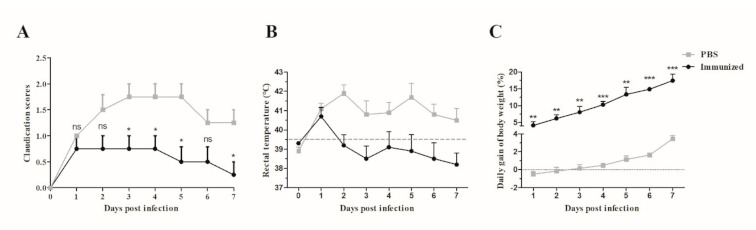
Clinical score (**A**), rectal temperature (**B**), and body weight (**C**) in the vaccinated piglets after the challenge with SS2. Signs of rectal temperature and body weight were monitored for 7 d post-infection. In (**A**), an unpaired Student’s *t*-test showed that the clinical score in the immunized piglets was significantly lower than in PBS-immunized piglets. In (**B**), recovery was indicated at a rectal temperature below 39.5 °C. In (**C**), an unpaired Student’s *t*-test showed that daily gain of body weight in the immunized piglets was significantly higher than in the PBS-immunized piglets. Samples collected from each piglet were tested in triplicate. *** *p* < 0.001, ** *p* < 0.01, * *p* < 0.05, and ns (*p* > 0.05). The error bars represent SEM.

No clinical signs of *S. suis* infection were observed in any of the piglets during the adaptation period prior to the challenge. The antibody titers against HA9801 strains or GAPDH, MRP, DLD, and JointS protein were determined in serum collected from both the immunized and the control piglets. The immunized group exhibited a strong antigen-specific immune response after the booster (Figure 4A). Furthermore, the specific antibody titers against GAPDH were significantly lower than MRP and DLD on the seventh day after the booster (Figure 4B). Two weeks after the boost immunization, all piglets were challenged by HA9801 strains. The piglets in the control group showed severe clinical signs of disease at one day post-infection, including fever (higher than 39.5 °C during 7 d, Figure 5A,B), anorexia, an obvious lack of coordination, and severe claudication; these clinical signs lasted for a period of five days. However, in the immunization group, the piglets showed mild clinical signs of disease (fever at two days post-infection) and they recovered quickly. Furthermore, the body weight of the piglets in the immunized group increased by 17.4% (from 5.85 to 6.87 kg), whereas only a 3.8% increase (from 6.11 to 6.34 kg) was observed in the control piglets after seven days post-infection (Figure 5C).

## 4. Discussion

Several antigenic proteins of *S. suis* have been tested as subunit vaccine candidates, but *S. suis* vaccine development is still ongoing [2,9]. Compared to intracellular proteins, extracellular and surface proteins of bacterial pathogens have the advantage of being easily recognized by the infected host and thus are candidates for vaccine development [10,30]. In the present study, three extracellular or surface proteins, MRP, GAPDH, and DLD, were tested. Previous studies found that MRP and GAPDH play an important role in nutrient transport, cellular metabolism, and virulence-related functions such as adhesion, invasion, and host defenses [11,15,19]. However, DLD is a novel potential immunogen in *S. suis* identified by immunoproteomic analysis in previous work, but the protective efficacy of DLD as a subunit vaccine was not clear [11]. DLD of *Pseudomonas aeruginosa* was found to be a surface-exposed protein that binds the human plasma proteins and may contribute to tissue invasion [31]. In addition, it was suggested that the DLD of *Vibrio* is involved in adhesion to cells and tissues of *Apostichopus japonicus* [32]. Although the previous findings showed that MRP, GAPDH, and DLD were potential immunogens, their protective efficacy still needs to be improved.

Two or more important antigenic proteins mixed or covalently linked together as a novel vaccine for bacterial infection have previously been proven to be much more effective than simple single antigenic proteins [1,10]. Therefore, in this study, three *S. suis* subunit vaccine candidates composed of recombinant composite proteins were developed, and their immune-protective efficacy was evaluated in zebrafish, mice, and swine by challenging them with a highly virulent SS2 HA9801, which is one of the most important *S. suis* pathotypes in China [15].

Reliable animal models are crucial for vaccine testing. It is known that zebrafish are an ideal animal model for vaccine research because of their innate and adaptive immune systems and the similarity of their nonspecific immunity system to that in mammals [33,34]. A large number of zebrafish can be used without the limitation of animal resources [25,35]. Therefore, recombinant proteins against different serotypes of *S. suis* offering good immunity could first be screened and identified in zebrafish, and then the preliminary results could be confirmed in host animals [36,37]. Our results found that, regardless of whether the zebrafish were infected with SS2, SS7, or SS9, the relative survival percentage for zebrafish immunized with the recombinant protein JointS was much higher than those immunized with GAPDH, MRP, or DLD, respectively. This demonstrates that three protective antigens linked together are more effective than any of the proteins individually.

In order to enhance the immune efficacy in vivo of recombinant protein JointS, it is necessary to select an effective and safe adjuvant to stimulate the immune response of the host. Most TLR-related agonists are potential candidates for swine vaccine adjuvants due to their ability to induce a pro-inflammatory cytokine response and immune cell activation [37,38,39]. However, there is no evidence that TLR-related agonists performed as an adjuvant combined with inactivated or subunit vaccines to prevent *S. suis* infections in pigs. It has been discovered that MPLA (TLR4 ligand) is a detoxified derivative of lipopolysaccharide (LPS), and R837 (Imiquimod) is a synthetic TLR7 ligand. Both of them have an immunomodulatory impact on the innate and adaptive immune system, and have been used as a safe and effective vaccine adjuvant in humans and animals [40,41,42]. However, our study found that a relatively low dose of MPLA induced high levels of innate immune-related cytokines (IFN-γ and IL-6) in the immune cells of mice and pigs, as well as in the serum of mice, indicating that MPLA stimulates the body more effectively to produce innate immune responses than R837. Furthermore, IFN-γ is a typical Th1-type cytokine that is effective for protection against intracellular infections, while the Th2 immune response is required for protective immunity against extracellular infections and is characterized by the production of IL-6 [40,41]. Particularly, the role of the Th2-type immune response is to help B cells to produce specific antibodies, which explains why, with the assistance of MPLA, JointS increased the titer of anti-*S. suis* antibodies and therefore all mice were protected from infection [42].

Previous studies have not shown a satisfactory protective effect in pigs with the useful subunit vaccine against *S. suis* infections in mice [8]. But our trivalent subunit vaccine also has good immune protection in piglets (Figure 4 and Figure 5). Unfortunately, there was no data on immune protection rates because no piglets died in the immunized or control groups after the challenge, though the clinical signs of disease of a typical *S. suis* infection partly reflected that the immunized piglets began to recover to healthy on the third day after infection (Figure 5). Furthermore, we observed that the specific antibody titers against individual MRP, DLD, or GAPDH proteins showed an unequal distribution, with the titers against GAPDH being significantly lower than MRP or DLD (Figure 4B). This implies that the protective effect induced by JointS was predominantly due to MRP and DLD, which also indicated that GAPDH may not play an important role in the induction of protective immunity against SS2 infection.

## 5. Conclusions

In this study, a recombinant trivalent protein JointS was identified and showed a good protection against virulent *S. suis* infections in zebrafish, mice or piglets. Furthermore, TLR4 agonists—MPLA can help JointS stimulate strong and persistent innate and acquired immune responses in vivo. Therefore, there is potential to develop JointS as a novel vaccine for piglets to prevent infection by SS2 and other *S. suis* serotypes.

## Figures and Tables

**Table 1:** Primers used for amplify *mrp*, *gapdh*, *dld*, and *gapdh*-*mrp*-*dld*.

Primer	Sequence (5′–3′) *^a^*	Restriction Site
P1	GTAGGATCCGAACAGGTAACATCAGA	*BamH* I
P2	GTACTCGAGCAAAGAGTAACGAATGTA	*Sac* I
P3	CAGGATCCGTAGTTAAAGTTGGTA	*BamH* I
P4	AAGAATTCTTTAGCGATTTTTGCG	*EcoR* I
P5	ACGAATTCATCAAAGGTCGTAGCA	*EcoR* I
P6	AACTCGAGGAACATCAAGAAAGGC	*Sac* I
P7	GTAGGATCCGAACAGGTAACATCAGA	*BamH* I
P8	TCCACCTCCACCTCCCAAAGAGTAACGAATGTA	
P9	GGAGGTGGAGGTGGAGTAGTTAAAGTTGGTA	
P10	TCCACCTCCACCTCCTTTAGCGATTTTTGCG	
P11	GGAGGTGGAGGTGGAATCAAAGGTCGTAGCA	
P12	AACTCGAGGAACATCAAGAAAGGC	*Sac* I

*^a^* Restriction sites are underlined.

**Table 2:** LD_50_ of the zebrafish after challenged by SS2.

CFU/Fish	Zebrafish No.	Death No.	Mortality (100%)
10^9^	15	15	100
10^8^	15	15	100
10^7^	15	14	93.3
10^6^	15	10	66.7
10^5^	15	6	40
10^4^	15	2	13.3
PBS	15	0	0
LD_50_	2.36 × 10^5^ CFU		

**Table 3:** LD_50_ of the zebrafish after challenged by SS7.

CFU/Fish	Zebrafish No.	Death No.	Mortality (100%)
10^9^	15	15	100
10^8^	15	15	100
10^7^	15	14	93.3
10^6^	15	8	53.3
10^5^	15	3	20.0
10^4^	15	1	6.7
PBS	15	0	0
LD_50_	7.96 × 10^5^ CFU		

**Table 4:** LD_50_ of the zebrafish after challenged by SS9.

CFU/Fish	Zebrafish No.	Death No.	Mortality (100%)
10^9^	15	15	100
10^8^	15	15	100
10^7^	15	11	73.3
10^6^	15	6	40.0
10^5^	15	1	6.7
10^4^	15	0	0
PBS	15	0	0
LD_50_	2.00 × 10^6^ CFU		

**Table 5:** Primers used for qRT-PCR.

Primer	Sequence (5′–3′)	Function
MIL − 6-F	TCCAGTTGCCTTCTTGGGAC	A fragment for murine IL − 6 gene
MIL − 6-R	GTGTAATTAAGCCTCCGACTTG	
MIL − 8-F	ATGGCTGCTGAACCAGTAGA	A fragment for murine IL − 8 gene
MIL − 8-R	CTAGTCTTCGTTTTGAACAG	
MIFN-γ-F	*AGCTCCCAGAAACTGAACGA*	A fragment for murine IFN-γ gene
MIFN-γ-R	*AGGGTTCAAAGCATGAATGG*	
PIL − 6-F	ACTGGCAGAAAACAACCTGA	A fragment for porcine IL − 6 gene
PIL − 6-R	CTAATCTGCACAGCCTCGAC	
PIL − 8-F	ATAAATACGCATTCCACACC	A fragment for porcine IL − 8 gene
PIL − 8-R	GTACAACCTTCTGCACCCA	
PIFN-γ-F	AGCCTCAATGACGACCTA	A fragment for porcine IFN-γ gene
PIFN-γ-R	ATCTTTGTTGGAGGGTGA	
MGAPDH-F	CCACAGTCCATGCCATCAC	A fragment for murine reference gene
MGAPDH-R	TCCACCACCCTGTTGCTGTA	
PGAPDH-F	GCTGGTGCTGAGTATGTCGT	A fragment for porcine reference gene
PGAPDH-R	AAGCAGTTGGTGGTACAGG	

## Data Availability

Data can be requested by writing to the authors.

## Supplementary Materials

The following are available online at https://www.mdpi.com/2076-393X/9/2/184/s1, Figure S1: SDS-PAGE and western blot analyses of GAPDH (A), MRP (B), DLD (C), and recombinant JointS (D) proteins. M, Maker; Lane 2, the SDS-PAGE analysis of purified proteins; Lane 3, the western blot analysis of purified proteins with convalescent sera against Streptococcus suis serotype 2 HA9801. Figure S2: Scheme of the vaccination protocols for (A) zebrafish, (B) mice, and (C) piglets.
