# Supplementary material for: TLR4 Agonist Combined with Trivalent Protein JointS of Streptococcus suis Provides Immunological Protection in Animals
Source: Vaccines (Basel). 2021 Feb 22;9(2):184. doi: 10.3390/vaccines9020184 (PMC7926372; doi:10.3390/vaccines9020184)
Supplement: Supplementary file 1 [file vaccines-09-00184-s001.pdf]

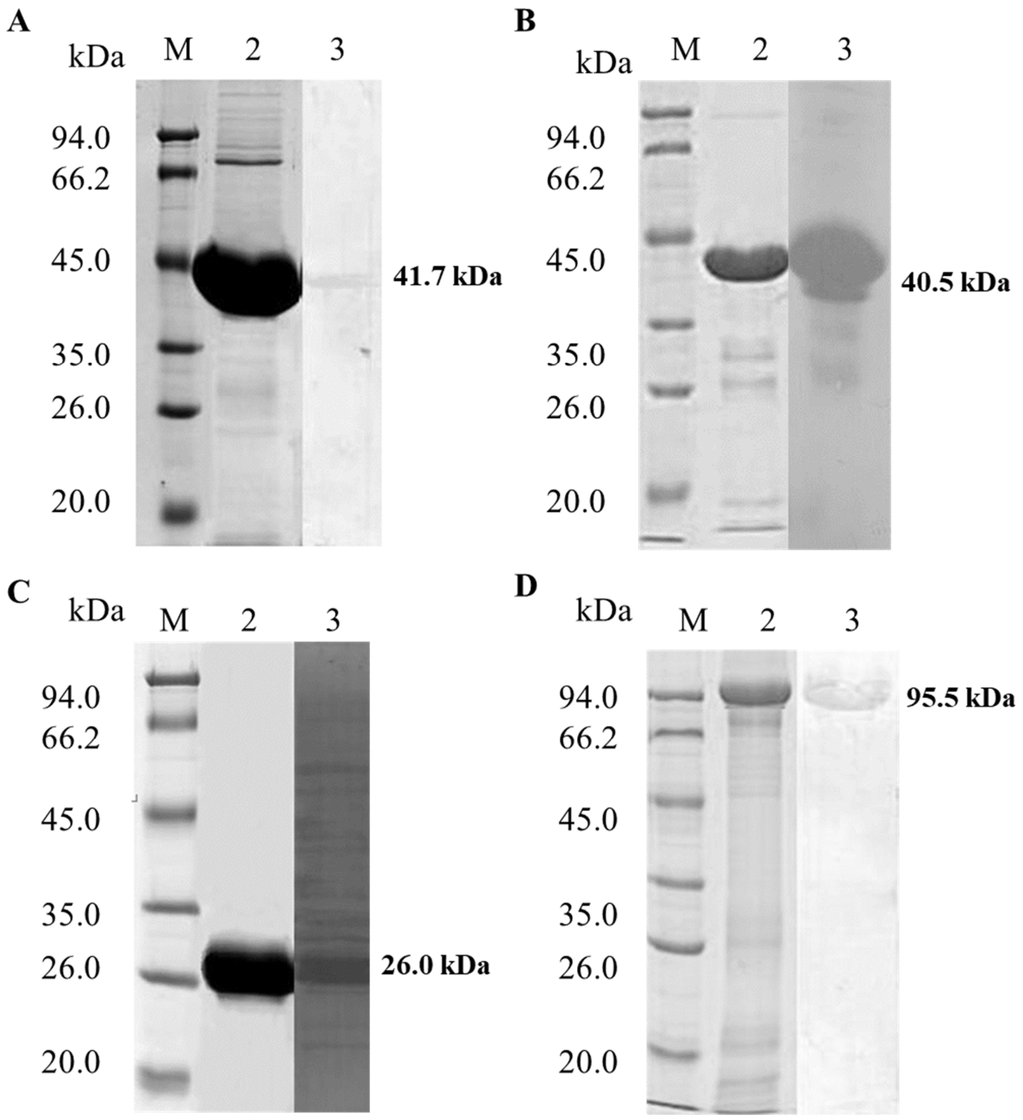

Figure S1 SDS-PAGE and western blot analyses of GAPDH (A), MRP (B), DLD (C), and recombinant JointS (D) proteins

M, Maker; Lane 2, the SDS-PAGE analysis of purified proteins; Lane 3, the western blot analysis of purified proteins with convalescent sera against *Streptococcus suis* serotype 2 HA9801.

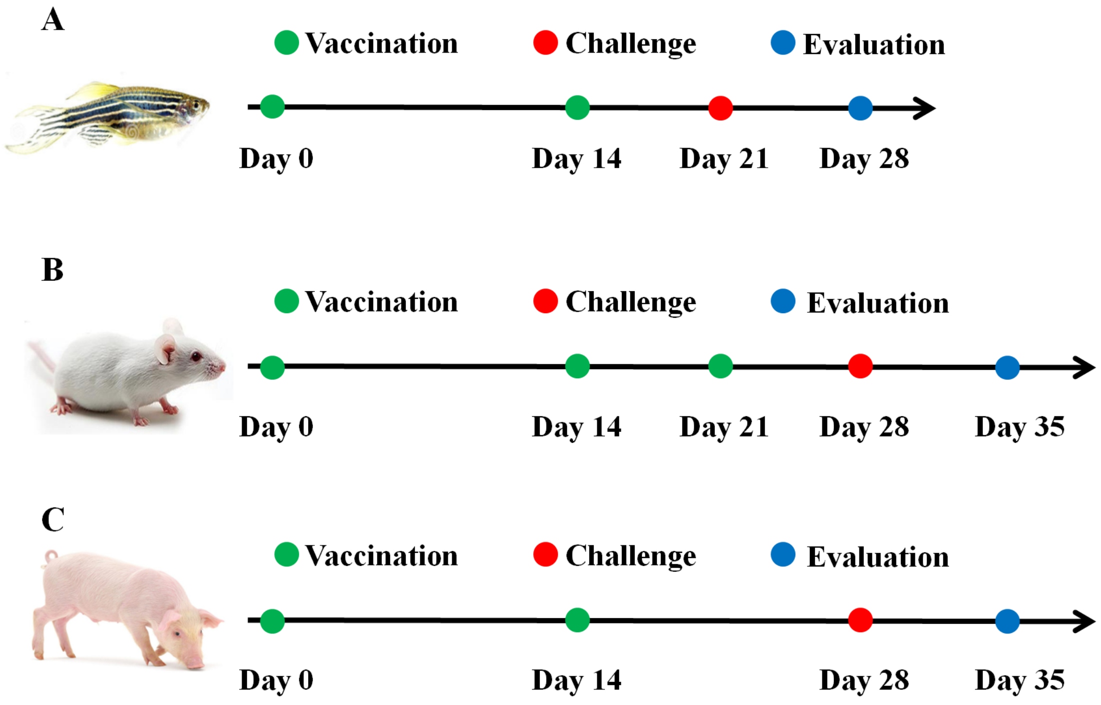

Figure S2 Scheme of the vaccination protocols for (A) zebrafish, (B) mice, and (c) piglets
